# Plant and soil responses to tillage practices change arbuscular mycorrhizal fungi populations during crop growth

**DOI:** 10.3389/fmicb.2024.1394104

**Published:** 2024-04-08

**Authors:** Jing Li, Lijuan Jia, Paul C. Struik, Zhengfeng An, Zhen Wang, Zhuwen Xu, Lei Ji, Yuqing Yao, Junjie Lv, Tao Zhou, Ke Jin

**Affiliations:** ^1^Institute of Grassland Research, Chinese Academy of Agricultural Sciences, Hohhot, China; ^2^School of Ecology and Environment, Inner Mongolia University, Hohhot, China; ^3^Department of Plant Sciences, Centre for Crop Systems Analysis, Wageningen University and Research, Wageningen, Netherlands; ^4^Department of Renewable Resources, University of Alberta, Edmonton, AB, Canada; ^5^Luoyang Academy of Agriculture and Forestry Sciences, Luoyang, China; ^6^Ningxia Academy of Agriculture and Forestry Sciences, Shizuishan, China

**Keywords:** tillage practices, arbuscular mycorrhizal fungi, wheat growth stages, co-occurrence, Loess Plateau

## Abstract

**Background:**

Tillage practices can substantially affect soil properties depending on crop stage. The interaction between tillage and crop growth on arbuscular mycorrhizal fungi (AMF) communities remains unclear. We investigated the interactions between four tillage treatments (CT: conventional tillage, RT: reduced tillage, NT: no tillage with mulch, and SS: subsoiling with mulch), maintained for 25 years, and two wheat growth stages (elongation stage and grain filling stage) on AMF diversity and community composition.

**Results:**

The AMF community composition strongly changed during wheat growth, mainly because of changes in the relative abundance of dominant genera *Claroideoglomus*, *Funneliformi*, *Rhizophagu*, *Entrophospora*, and *Glomus*. Co-occurrence network analysis revealed that the grain filling stage had a more complex network than the elongation stage. Redundancy analysis results showed that keystone genera respond mainly to changes in soil organic carbon during elongation stage, whereas the total nitrogen content affected the keystone genera during grain filling. Compared with CT, the treatments with mulch, i.e., NT and SS, significantly changed the AMF community composition. The change of AMF communities under different tillage practices depended on wheat biomass and soil nutrients. NT significantly increased the relative abundances of *Glomus* and *Septoglomus*, while RT significantly increased the relative abundance of *Claroideoglomus.*

**Conclusion:**

Our findings indicate that the relative abundance of dominant genera changed during wheat growth stages. Proper tillage practices (e.g., NT and SS) benefit the long-term sustainable development of the Loess Plateau cropping systems.

## 1 Introduction

Agricultural practices affect not only crop production (Singh et al., 2016) but also soil quality (Zhang et al., 2015). Excessive tillage practices, as common in conventional tillage, increase the bare soil area, intensify soil erosion, destroy soil structure, and reduce soil organic matter, resulting in a yield decline (Kabiri et al., 2016; Yan et al., 2020). In order to maintain the sustainable development of agriculture, improving nutrient use efficiency is crucial to increase crop yields (Yan et al., 2020). Thus, many studies have focused on increasing crop yield through conservation tillage practices (e.g., no tillage, reduced tillage, and subsoiling with mulch) (Jin et al., 2008; Chen et al., 2019; Jia et al., 2022). Conservation tillage practices with minimal soil disturbance improve agricultural sustainability by reducing soil erosion and enhancing the diversity of microorganisms (Zuber et al., 2015; Xiao et al., 2019). Among them, arbuscular mycorrhizal fungi (AMF), as important rhizosphere microorganisms, were affected by tillage practices (Balota et al., 2016; Oehl and Koch, 2018). However, the linkages between the responses of plant–soil-AMF to conservation tillage practices, especially the timing and duration of tillage practices are still unclear.

AMF can create symbioses with the roots of more than 80% of terrestrial plant species, which enhances the ability of plants to overcome the adverse impacts of and to ameliorate plant performance under environmental stress (such as drought, extreme temperature and changes in land management) (Walder and van der Heijden, 2015; Gu et al., 2020). The mechanisms of vascular plants to resist environmental change involve forming symbiotic relationships with AMF, which allows the AMF to acquire plant-synthesized carbon (C) as well as enhance the nutrient supply (mainly N and P) to the host plants as shown by many previous studies (e.g., Augé et al., 2015; Wang et al., 2017). Conventional tillage can decrease the diversity and activity of AMF, which negatively affects the symbiotic relationship between AMF and plants and results in the decline of crop yield and soil quality (Jansa et al., 2003; Avio et al., 2013). For example, long-term conventional tillage decreased the AMF richness and induced a marked shift in the community composition by changing the functional quality of AMF (e.g., spore density or hyphal networks) (Schnoor et al., 2011; Säle et al., 2015).

Conservation tillage (reduced tillage, no tillage, and subsoiling with mulch) is considered to be an effective technology improving the stability of soil and AMF attributes, which can help maintain a more complex interaction network and increase the resilience of soil to interference (Carballar-Hernández et al., 2017; Gu et al., 2020). For instance, no tillage enhanced AMF activity and diversity by improving soil fertility, storing and conserving water, and reducing soil erosion (Bowles et al., 2017). Subsoiling with mulch also increased AMF activity and diversity by stimulating water infiltration and increasing soil carbon and nitrogen concentrations (Gu et al., 2020; Wang Z. et al., 2020; Wang S. L. et al., 2020). However, long-term conservation tillage, e.g., no tillage, may lead to problems such as soil surface hardening and more limited O_2_ supply for soil organisms, which may be detrimental to the distribution of AMF propagules (Curaqueo et al., 2011). Although previous studies have shown that different tillage practices alter the composition of AMF communities, the direction and extent of the impact of different tillage practices on the composition of AMF communities are highly uncertain.

At different stages of crop growth, the community composition of AMF presents a dynamic change process (Hu et al., 2015; Wang Z. et al., 2020; Wang S. L. et al., 2020). Plant roots recruit rhizosphere AMF by secreting and absorbing different chemicals, leading to a shift in the rhizosphere AMF community across the different crop growth stages (Edwards et al., 2015; Hamonts et al., 2018). In addition, the interaction between the host and AMF regulates the growth of the AMF community in the root system (Yang et al., 2015). For example, during different phenological stages, the colonization by AMF of the plant roots changes, further affecting the community composition of AMF across crop growth stages (Merryweather and Fitter, 1998; Schalamuk et al., 2004). At present, most studies focus on the effect of tillage practices or crop growth stages on the community composition of AMF, while few studies investigate the interaction of tillage practices and crop growth stages to mediate AMF communities (Hu et al., 2015). Therefore, further research is essential to assess the temporal dynamics of AMF communities and the impact of tillage practices on AMF communities throughout crop growth and development.

As one of the largest and most important food crops in the Loess Plateau, winter wheat (*Triticum aestivum* L.) plays an important role in food security and contributes more than 70% of the agricultural production in northern China (Xue et al., 2019; Xia et al., 2020). Our previous studies have found that tillage practices affect winter wheat production, soil physicochemical properties, and microbial community composition (Jin et al., 2008; Jia et al., 2022). However, there is little information about the impact of tillage practices on the species richness and composition of AMF in the winter wheat production systems, especially at different winter wheat growth stages. Here, we assessed a 22-year field experiment examining the potential impacts of four contrasting tillage practices at two growth stages (elongation and grain filling) on AMF dynamics in the semi-arid agricultural ecosystem. We hypothesized that (1) long-term tillage practices create particular populations of AMF by altering the species diversity of AMF and the abundance of keystone operational taxonomic units or genera; (2) the effect of long-term tillage practices on AMF communities relies on the shifts in the rhizosphere environment and on biomass accumulation during winter wheat production; and (3) conservation tillage can develop particular groups of AMF by enhancing soil fertility and winter wheat productivity.

## 2 Materials and methods

### 2.1 Study sites and experimental design

The experiment started in 1999 in the eastern part of the Loess Plateau, Songzhuang Village (34°58′N, 113°08 E), Henan Province, China. The area had previously been conventionally tilled for more than 30 years before the experimental plot was established. The thickness of the Quaternary loess layer is 50 to 100 m in this area. The soil in the study area is silt loam and is classified as Inceptisol according to soil taxonomy (Soil Survey Staff, 2003). The basic soil properties were analyzed to assess plot homogeneity based on their contribution to crop performance and soil quality in previous studies (Jin et al., 2009).

Four tillage practices, conventional tillage (CT), no tillage with mulch (NT), reduced tillage (RT), and subsoiling with mulch (SS), were set up at four nearby sites. For example, for the CT treatment, there was still 10–15 cm of stubble after harvest (May 25 to June 1), but the straw and ears had been removed from the site at harvest. In the first week of July, the soil was turned to a depth of 20 cm. Around October 1, before winter wheat was sown, the soil was turned again to a depth of 20 cm, fertilizer was mixed in, and then the soil was harrowed to prepare the seedbed. Winter wheat sowing occurred around October 5. For the RT treatment, 10–15 cm of stubble was left in the field after winter wheat harvest (May 25 to June 1), and the straw was returned to the field after harvest. Around July 15, deep plowing (25–30 cm) was combined with harrowing (5–8 cm) and compaction with a roller. Winter wheat was sown directly around October 5. Therefore, this practice only required one plowing instead of two under the CT. For the NT treatment, 30 cm of stubble was left in the field after harvest (May 25 to June 1), and the straw was returned to the field after threshing. Fertilization was carried out from September 25 to October 5. For the SS treatment, 25–35 cm of stubble remained in the field after harvest (May 25–June 1). Around July 1, subsoiling was carried out at 60 cm intervals to a depth of 30 to 35 cm. Sowing with fertilizer application was performed from September 2 to October 5. The set-up of the experiment was subject to pseudo-replication due to space-for-time substitution limitations (Blois et al., 2013). However, the four experimental sites appeared topographically similar and generally suffered little disturbance in previous studies regarding frequency and severity (Jia et al., 2022). Therefore, the tillage practices were considered the only important change factor across sites.

### 2.2 Plant and soil sampling

Winter wheat biomass and soil samples were collected on March 28 and May 20 (i.e., at the elongation and grain filling stages of winter wheat) in 2023. We randomly established six plots (0.5 × 0.5 m) in winter wheat crops grown in each tillage treatment. Twenty winter wheat plants were sampled and combined into one sample for each plot. The roots of winter wheat were collected from 0 to 20 cm in each plot. We shook all roots of each plot vigorously to remove soil that was not tightly adhered and used a sterile scalpel to separate the rhizosphere soil from the root surface carefully (Song et al., 2007; Liu et al., 2022). Two soil cores with a diameter of 7.5 cm were collected and then mixed with one soil sample (0–20 cm layer) per plot; thus, six AMF soil samples were collected for each treatment. A total of 48 AMF soil samples were collected (two growth stages × four tillage treatments × six AMF soil samples/tillage treatment).

### 2.3 Sample processing and measuring biotic and abiotic variables

Twenty winter wheat plants from each plot were separated into above-ground biomass and below-ground biomass, oven-dried at 65°C for 48 h and weighed to assess dry biomass (Jia et al., 2022). Each composite soil sample from each plot was divided into three subsamples. One subsample was air-dried to determine physicochemical properties. The second subsample was stored at 4°C and analyzed within a week to measure soil ammonium (NH_4_^+^) and nitrate (NO_3_^−^) content, microbial C and N biomass. The third subsample was immediately stored at −80 °C for DNA extraction and Miseq sequencing analysis within two days. The gravimetric method was used to measure soil water content (SWC). Soil pH was measured using a 1:2.5 soil: water mixture. The dichromate oxidation method was used to determine the soil organic carbon (SOC) (Nelson and Sommers, 1983), and total nitrogen (TN) was measured using an Elemental Analyzer (vario MACRO cube, Elementar, Germany). Soil NO_3_^−^ and NH_4_^+^ were extracted from the soil samples using a 2 mol L^−1^ KCl solution and were measured using a continuous flow analyzer (Auto Analyzer 3, Seal, Germany). Soil dissolved organic carbon (DOC) was measured using a TOC-L-ASI analyzer (Analytik Jena AG, Jena, Germany) by adding 0.5 M K_2_SO_4_ and shaking for 1 h. The total P (TP) concentration was determined using an Astoria auto-analyzer (Clackamas, OR). The available phosphorus (AP) content was determined using the Olsen method. Soil microbial biomass C (MBC) and N (MBN) were measured using fumigation extractions (Vance et al., 1987).

### 2.4 AMF root colonization

Fifty fine root fragments (approximately 1 cm long) from each sample were stained with trypan blue, and the percentage of AM root colonization was measured by the line intersection method at 200-fold magnification (McGonigle et al., 1990). Total AM root colonization was determined as the percentage of root length colonized by AM fungi.

### 2.5 DNA extraction and high-throughput sequencing

DNA Extraction Total genomic DNA samples were extracted from 0.5 g of each soil sample using the OMEGA Soil DNA Kit (M5635-02) (Omega Bio-Tek, Norcross, GA, United States), following the manufacturer’s instructions. The quantity and quality of extracted DNAs were measured using a NanoDrop NC2000 spectrophotometer (Thermo Fisher Scientific, Waltham, MA, United States) and agarose gel electrophoresis, respectively.

PCR amplification of the AMF genes region performed using the forward primer AMV4.5NF (5′-AAGCTCGTAGTTGAATTTCG-3′) AMDGR (5′-CCCAACTATCCCTATTAATCAT-3′) (Van Geel et al., 2014; Suzuki et al., 2020). Sample-specific 7-bp barcodes were incorporated into the primers for multiplex sequencing. The PCR components contained 5 μL of buffer (5×), 0.25 μL of Fast pfu DNA Polymerase (5 U/μl), 2 μL (2.5 mM) of dNTPs, 1 μL (10 uM) of each Forward and Reverse primer, 1 μL of DNA Template, and 14.75 μL of ddH_2_O. Thermal cycling consisted of initial denaturation at 98°C for 5 min, followed by 30 cycles consisting of denaturation at 98°C for 30 s, annealing at 53°C for 45 s, and extension at 72°C for 45 s, with a final extension of 5 min at 72°C. PCR amplicons were purified with Vazyme VAHTSTM DNA Clean Beads (Vazyme, Nanjing, China) and quantified using the Quant-iT PicoGreen dsDNA Assay Kit (Invitrogen, Carlsbad, CA, United States). After the individual quantification step, amplicons were pooled in equal amounts, and pair-end 2 × 250 bp sequencing was performed using the Illlumina NovaSeq platform with NovaSeq 6000 SP Reagent Kit (500 cycles) at Shanghai Personal Biotechnology Co., Ltd. (Shanghai, China).

Sequence data analyses were mainly performed using QIIME2. After quality filtering and removing chimeras, high-quality sequences were also clustered into operational taxonomic units (OTUs) using USEARCH at 97% sequence identity (v2.13.4).^1^ We selected one representative sequence for each OTU and aligned all representative sequences using PyNAST (Panneerselvam et al., 2020) with a minimum identity threshold of 0.8. The sequences were assigned to virtual taxa using the MaarjAM database (Koljalg et al., 2013).

### 2.6 Co-occurrence network construction and analyses

The “microeco” package was used to construct microbial ecological networks (Liu et al., 2021). We created the microtable class and performed basic preprocessing operations. The operational taxonomic unit (OTU) table was reduced so that sequence numbers were the same between samples (10,000 sequences in each sample). We calculated the taxa abundance for the downstream analysis. In a network, betweenness centrality (BC) calculates the fraction of the shortest paths through a given microbial taxon to another microbial taxon, which reflects the degree of control that a taxon exerts over the interactions between other taxa in the network (Lupatini et al., 2014). A valid interaction event is determined to have a robust correlation if the Pearson correlation coefficient is equal to or greater than 0.8 or equal to or lower than −0.8 and is statistically significant (*p*-value equal to or smaller than 0.05) (Liu et al., 2021). The gephi interactive platform^2^ was used to explore and visualize the network structure. Undirected networks and Fruchterman-Reingold are used for layout.

### 2.7 Statistical analyses

Statistical analyses of differences between treatments were conducted using R (v. 3.2.0). Two-way ANOVAs were used to examine the effects of two growth stages and four tillage practices on the winter wheat biomass (AB and BB), soil properties (pH, SWC, SOC, TN, TP, DOC, NH_4_^+^, NO_3_^−^, and AP) and microbial C (MBC) and N (MBN). Microbial diversity was examined using diversity metrics to analyze species diversity with QIIME, including Chao1, Observed_species, PD_whole, and Shannon’s diversity. Chao1 and Observed species indices represent richness, and Shannon indices represent diversity. Tukey’s HSD test was used to test pairwise comparisons. The statistically significant difference was defined at *p* < 0.05.

AMF community composition across the treatments was evaluated by principal coordinate analyses (PCoA) based on the Bray-Curtis distance. Permutational multivariate analyses of variance (PERMANOVA) with the “adonis” function in the “vegan” package were performed to examine the effect of growth stage and tillage treatment on AMF community composition. Correlation analysis was performed between each biotic and abiotic variable and AMF community composition at the OTU level (Sunagawa et al., 2015). Spearman correlations were used to examine the relationship between the abundance of dominant genera and each biotic and abiotic variable. Redundancy analysis (RDA) was used to visualize the relationships between environmental variables and soil microbial communities. The “envfit” function in the “vagan” package was used to test the correlation between each biotic and abiotic variable and AMF communities to determine the important environmental variables that can explain the variation in the composition of AMF communities (Lavergne et al., 2020).

## 3 Results

### 3.1 Wheat biomass and soil properties

Compared with the elongation stage, AB, BB, soil TN content, soil MBC and MBN content all were significantly higher in the grain filling stage (*p* < 0.001); The SOC decreased significantly in the grain filling stage (*p* = 0.002; Table 1). Compared with CT, the NT and SS treatments (both including mulching) were associated with a significant increase in AB, BB, soil pH, SOC, soil TN content, DOC, and soil MBC content, but with a significant reduction in soil AP content (*p* < 0.05; Table 1). The soil NO_3_^−^ content and soil MBN content were higher only in the NT treatment than in the CT treatment (*p* < 0.05; Table 1).

**Table 1:** Wheat above-ground biomass (AB), below-ground biomass (BB), 0–20 cm soil gravimetric water content (SWC), soil pH value, soil organic carbon content (SOC), soil total nitrogen content (TN), soil total phosphorus content (TP), soil NH_4_^+^ and NO_3_^−^ content, soil dissolved organic carbon (DOC), soil available phosphorus content (AP), soil microbial C (MBC) and N (MBN) biomass in response to two crop growth stages and tillage practices.

	Stage		Tillage practices				*p*-value		
	Elongation	Grain filling	CT	RT	NT	SS	S	T	S × T
AB (g m^−2^)	137b	246a	162b	168b	214a	222a	**<0.001**	**<0.001**	0.540
BB (g m^−2^)	45.4b	56.8a	42.0b	44.4b	57.2a	60.8a	**<0.001**	**<0.001**	0.256
SWC (%)	6.00	6.27	5.57b	6.38ab	6.72a	5.74ab	0.415	**0.007**	0.986
pH	8.13	8.19	7.98b	8.17a	8.27a	8.23a	0.098	**<0.001**	0.375
SOC (g kg^−1^)	8.40a	7.72b	7.27b	6.22b	9.02a	9.74a	**0.002**	**<0.001**	0.526
TN (g kg^−1^)	0.78b	0.83a	0.72b	0.63c	0.91a	0.96a	**0.003**	**<0.001**	**0.046**
TP (g kg^−1^)	1.02	1.06	0.98b	0.86c	1.12a	1.19a	0.181	**<0.001**	0.253
AP (mg kg^−1^)	22.48	22.17	28.58a	21.05b	18.27b	21.42b	0.832	**<0.001**	0.472
NO_3_^−^ (mg kg^−1^)	3.56	3.92	3.26b	3.42b	4.41a	3.87ab	0.138	**0.005**	0.788
NH_4_^+^ (mg kg^−1^)	1.91	2.38	2.52	2.37	2.22	1.47	0.093	0.056	0.845
DOC (mg kg^−1^)	17.7	16.9	11.0b	9.7b	21.9a	26.6a	0.297	**<0.001**	**0.001**
MBC (mg kg^−1^)	89.2b	264.5a	168.0c	117.2d	198.6b	223.7a	**<0.001**	**<0.001**	**0.001**
MBN (mg kg^−1^)	9.97b	14.49a	8.18c	12.53b	19.30a	8.91c	**<0.001**	**<0.001**	**<0.001**

Bold values are significant (*p* < 0.05). S is crop stage effect; T is tillage treatment effect.

For each parameter, data in a row followed by a different lowercase letter indicates significant difference at the 0.05 probability level based on protected HSD tests.

Values for elongation stage and grain filling stage are averaged across tillage treatments (*n* = 24).

Values for tillage practices are averaged across crop stages (*n* = 12). CT, conventional tillage; NT, no tillage with mulch; RT, reduced tillage; SS, subsoiling with mulch.

### 3.2 Variation in soil AMF communities

AMF root colonization was significantly influenced by the growth stage (*F* = 10.87, *p* = 0.002) and tillage practice (*F* = 58.47, *p* < 0.001; Supplementary Figure S1). Root colonization by AMF was significantly more advanced at the grain filling stage than at the elongation stage (*p* < 0.05; Supplementary Figure S1). AMF root colonization was stronger in both NT and SS (the two tillage treatments with mulch) than in CT at both growth stages (*p* < 0.05; Supplementary Figure S1).

**Figure 1:**
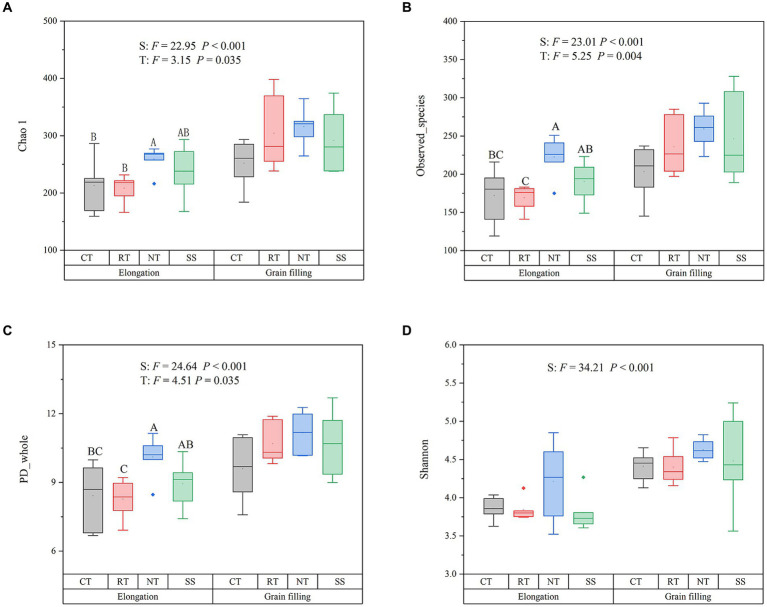
Responses of the alpha diversity of AMF to different tillage practices at two growth stages. Chao 1 **(A)**; Observed_species **(B)**; PD_whole **(C)**; Shannon **(D)**. Results are reported as the mean ± standard error (*n* = 6). For each parameter, a different letter indicates a significant difference at the 0.05 probability level (*p* < 0.05) based on Tukey’s honest test. CT, conventional tillage (*n* = 6); NT, no tillage with mulch (*n* = 6); RT, reduced tillage (*n* = 6); SS, subsoiling with mulch (*n* = 6).

AMF alpha diversity, as measured by the Chao1 (*F* = 22.95, *p* < 0.001), the Observed_species (*F* = 23.01, *p* < 0.001), PD_whole (*F* = 24.64, *p* < 0.001), and Shannon diversity indexes (*F* = 34.21, *p* < 0.001), was significantly higher in the grain filling stage than in the elongation stage (Figure 1). The Chao1, the Observed_species, and PD_whole index were higher in the NT treatment than in the CT treatment at elongation (*p* < 0.05; Figure 1).

**Figure 2:**
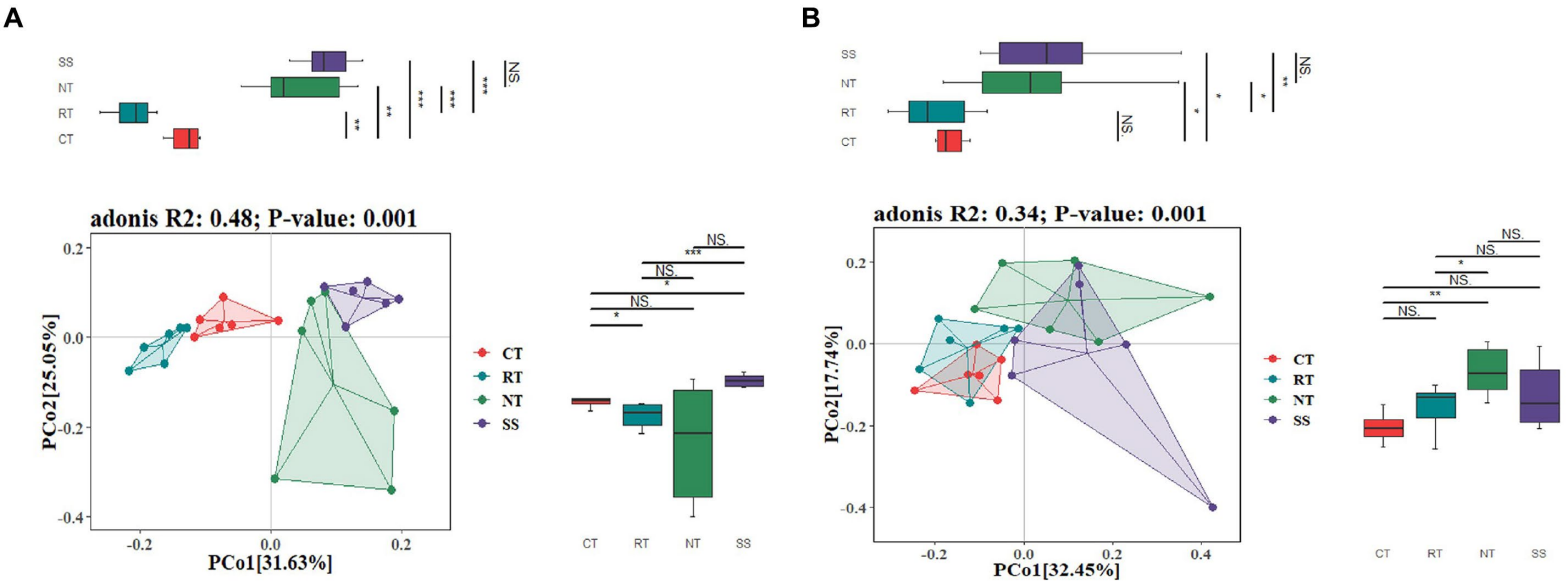
Principal coordinate analysis (PCoA) of the dissimilarity of AMF based on the Bray–Curtis distance at elongation stage **(A)** and grain filling stage **(B)**. CT, conventional tillage (*n* = 6); NT, no tillage with mulch (*n* = 6); RT, reduced tillage (*n* = 6); SS, subsoiling with mulch (*n* = 6).

The PCoA ordination showed that the AMF community composition was clearly different among different tillage practices at elongation based on the Adonis (PERMANOVA) test (*R*^2^ = 0.48, *p* < 0.001; Figure 2A). CT was clearly separated from NT and RT (Figure 2A). Similarly, the PCoA also showed that the AMF community composition was clearly different among different tillage practices for the grain filling stage based on the Adonis (PERMANOVA) test (*R*^2^ = 0.34, *p* < 0.001; Figure 2B). CT was similar to RT, but was clearly separated from NT and SS (Figure 2B).

**Figure 3:**
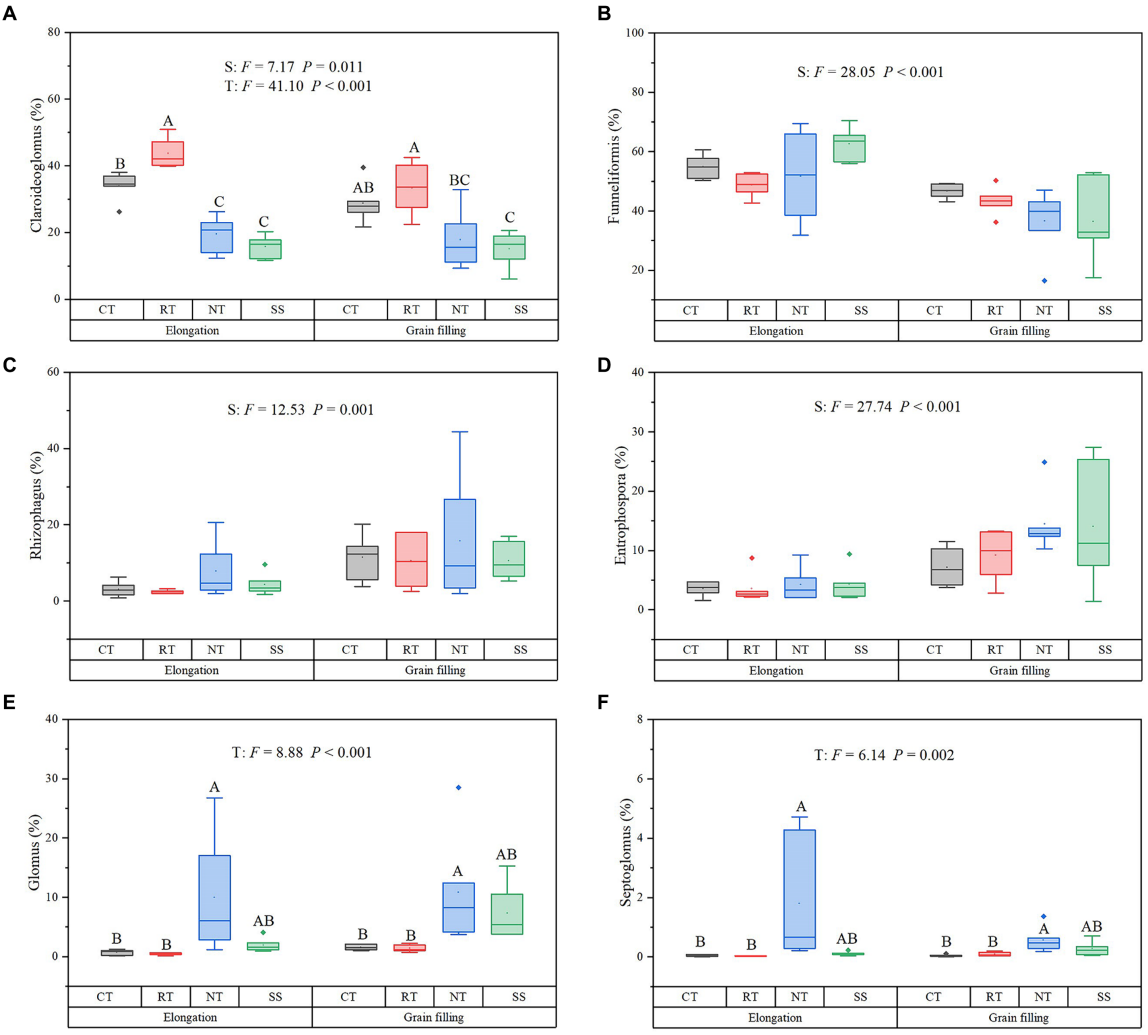
Response of dominant genera to different tillage practices at two growth stages. For each parameter, a different letter indicates a significant difference at the 0.05 probability level (*p* < 0.05) based on Tukey’s honest test. Shown are the dominant genera: *Claroideoglomus* **(A)**, *Funneliformis* **(B)**, *Rhizophagus* **(C)**, *Entrophospora* **(D)**, *Glomus* **(E)**, and *Septoglomus* **(F)**. CT, conventional tillage (*n* = 6); NT, no tillage with mulch (*n* = 6); RT, reduced tillage (*n* = 6); SS, subsoiling with mulch (*n* = 6).

During winter wheat development, the following changes occurred in four dominant genera: the relative abundance of *Claroideoglomus* (*p* = 0.011), *Rhizophagus* (*p* = 0.001), and *Entrophospora* (*p* < 0.001) increased, whereas the relative abundance of *Funneliformis* decreased (*p* < 0.001; Figure 3). Compared with the CT treatment, the RT treatment significantly increased the relative abundance of *Claroideoglomus*, while NT and SS decreased the relative abundance of *Claroideoglomus* (*p* < 0.05; Figure 3). NT significantly increased the relative abundance of *Glomus* and *Septoglomus* (*p* < 0.05; Figure 3).

**Figure 4:**
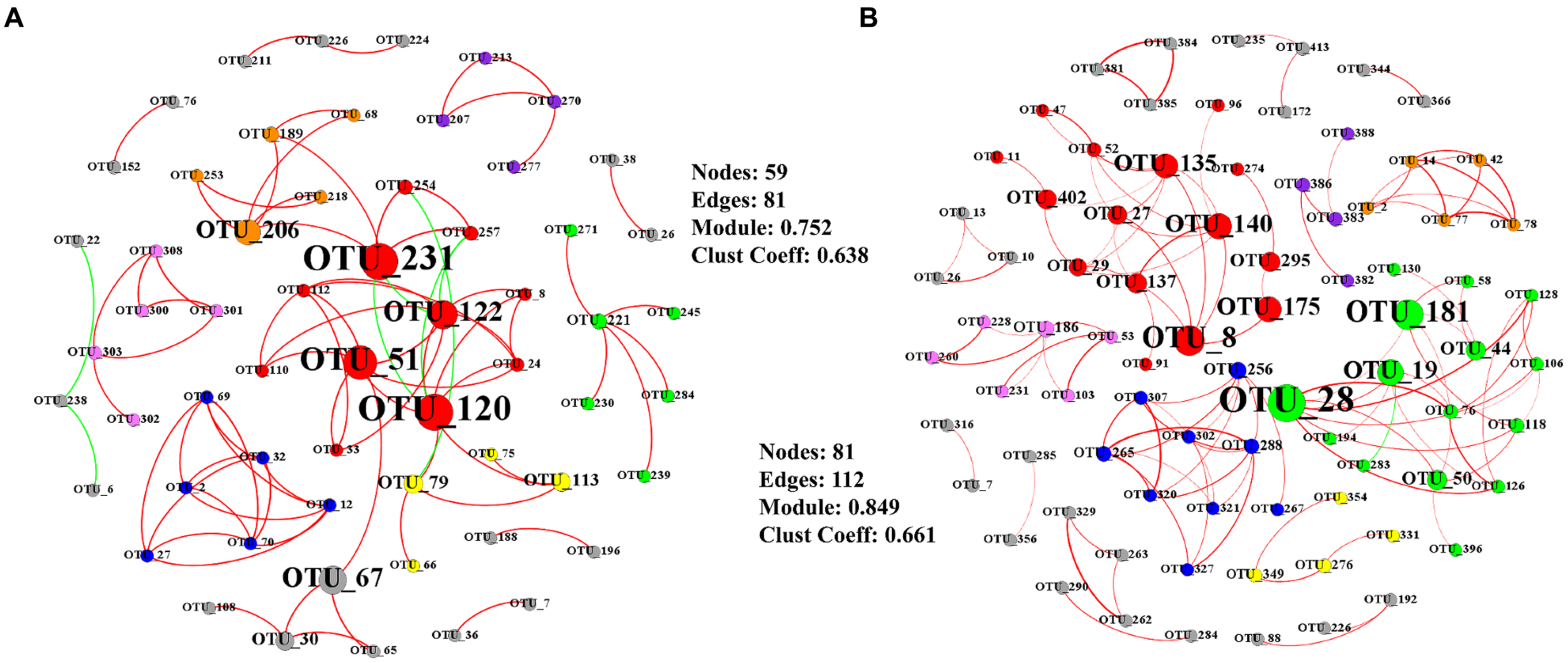
Co-occurrence networks of AMF at elongation stage **(A)** and grain filling stage **(B)**. Only Pearson’s correlation coefficients (r > 0.8 or r < − 0.8 significant at *p* < 0.05) are shown. Node sizes are proportional to the value of betweenness centrality. Red edges represent positive correlations and green edges represent negative correlations. The colors of the nodes represent the AMF modules.

Key taxa were shown based on the high betweenness centrality score of the network (Figure 4). The total AMF network was composed of 59 nodes and 81 significant edges at elongation (Figure 4A). The clustering coefficient and the average number of neighbors was 0.752 and 0.638, respectively (Figure 4A). The core groups included the OTU_120, OTU_51, OTU_122, and OTU_67 belonging to the genus *Glomus* (Figure 4A). Another taxon (OUT_231) belonged to the genus *Claroideoglomus*. The total AMF network consisted of 81 nodes, and 112 significant edges were detected at grain filling (Figure 4B). The clustering coefficient and the average number of neighbors were 0.849 and 0.661, respectively (Figure 4B). OTU_8, OTU_19, OTU_140, and OTU_175 were characterized as keystone taxa, which belonged to the genus *Glomus* (Figure 4B). OTU_28 belonged to genus *Rhizophagus* (Figure 4B).

### 3.3 AMF community composition was related to wheat biomass and soil environmental variables

**Figure 5:**
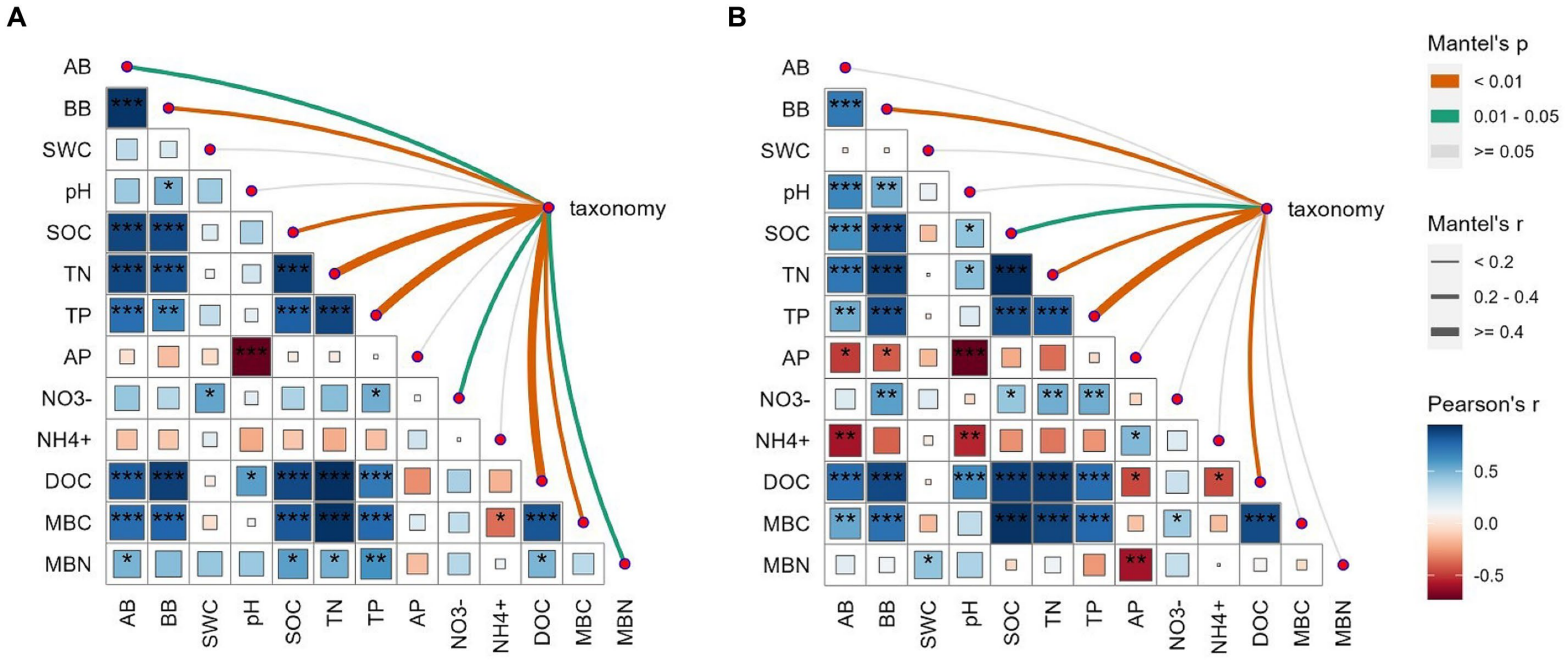
Correlation analysis between measured variables (wheat biomass and soil physicochemical properties) and AMF community composition at the OTU level under elongation stage **(A)** and grain filling stage **(B)**. Circle colors and sizes represent Spearman’s correlation coefficient (r) values, while *, **, and *** indicate statistically significant differences at *p* < 0.05, 0.01 ≤ *p* ≤ 0.05, and *p* < 0.01, respectively. The color and edge widths of the association lines represent Mantel r values, while red and gray lines represent *p* < 0.05 and *p* > 0.05 statistical significance values, respectively.

**Figure 6:**
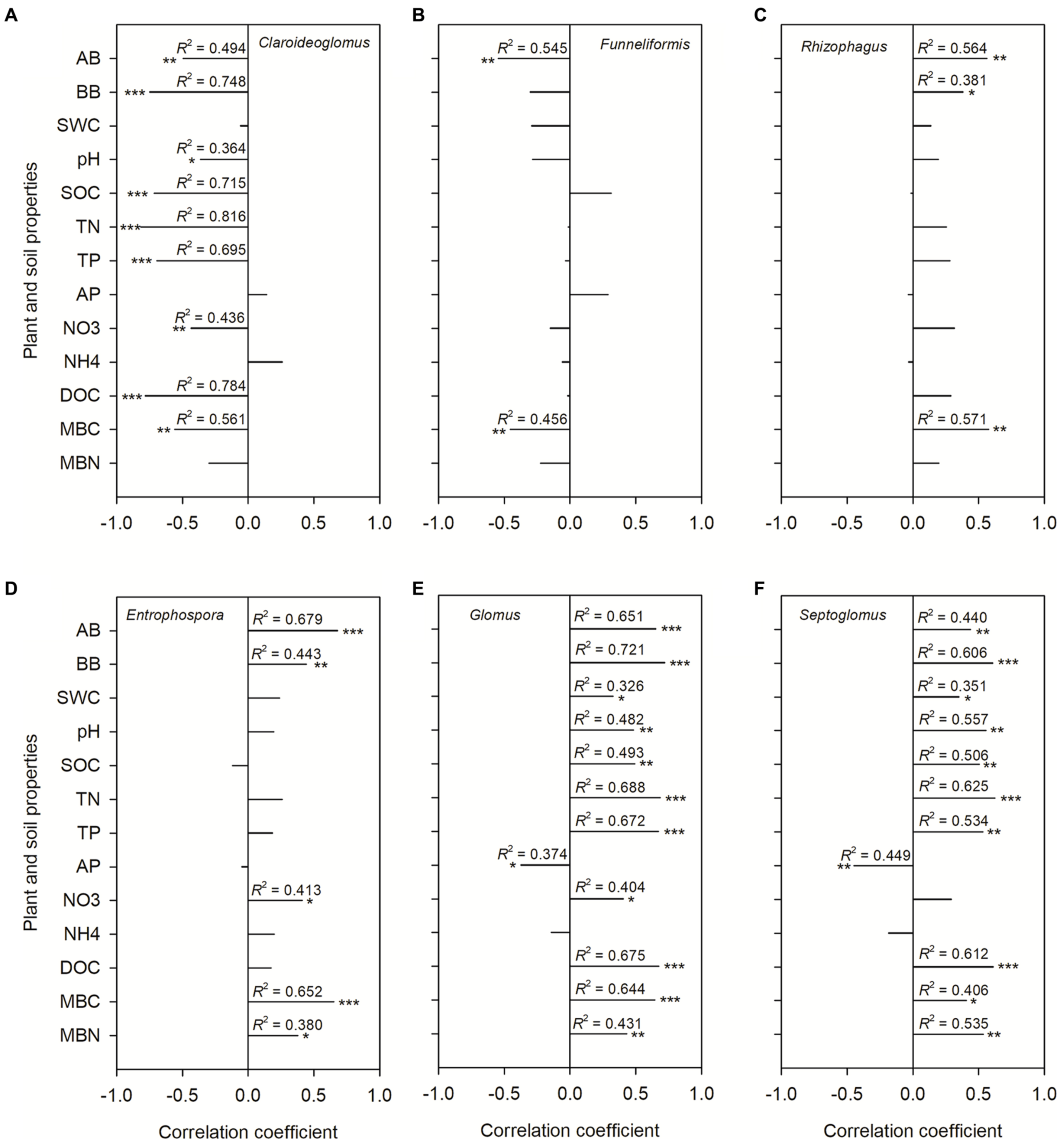
The Spearman correlation coefficients between dominant genera and plant and soil properties. Shown are the dominant genera: *Claroideoglomus* **(A)**, *Funneliformis* **(B)**, *Rhizophagus* **(C)**, *Entrophospora* **(D)**, *Glomus* **(E)**, and *Septoglomus* **(F)**. The correlations were derived for above-ground biomass (AB), below-ground biomass (BB), 0–20 cm soil gravimetric water content (SWC), soil pH value, soil organic carbon content (SOC), soil total nitrogen content (TN), soil total phosphorus content (TP), soil NH4 (NH_4_^+^) and NO3 (NO_3_^−^) content, soil dissolved organic carbon (DOC), soil available phosphorus content (AP), soil microbial C (MBC) and N (MBN) biomass.

**Figure 7:**
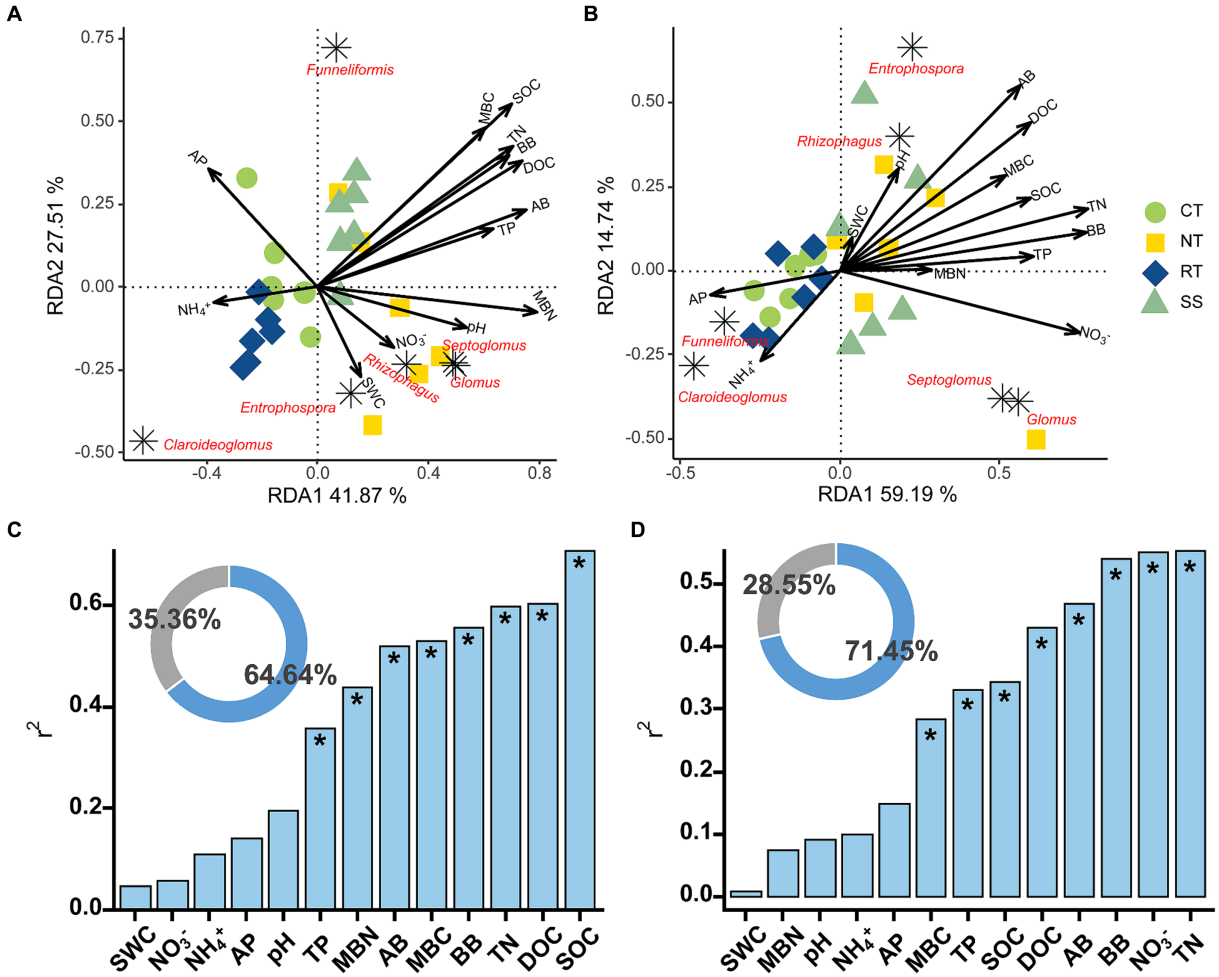
Redundancy analysis (RDA) of AMF dominant genera and environmental variables at elongation stage **(A)** and grain filling stage **(B)**. The Envfit function was used to identify important environmental variables associated with order under elongation stage **(C)** and grain filling stage **(D)**. The blue part of the circle diagram represents the degree of explanation of community composition differences by environmental variables, while the gray part represents the part of community structure differences that are unexplained by environmental variables. Significance level is as follows: *p < 0.05.

The AMF communities were influenced by winter wheat biomass and soil properties (Figure 5). BB, SOC, TN, TP, and DOC were highly correlated with AMF community composition (Figure 5). Spearman correlation showed that significant negative correlations were found for the relative abundance of dominant genera (e.g., *Claroideoglomus* and *Funneliformis*) and plant and soil properties, whereas positive relationships were found between dominant genera (e.g., *Glomus* and *Rhizophagus*) and plant and soil properties (Figure 6). The redundancy analysis (RDA) showed significant correlations between winter wheat biomass, soil properties and dominant genera under tillage practices at different growth stages (*p* < 0.05; Figure 7). For dominant genera, the RDA1 and RDA2 explained 41.9 and 27.5% of the variance at the elongation stage, respectively (Figure 7A). At grain filling, the first two axes of the RDA analysis explained 73.9% of the total variance, with RDA1 and RDA2 explaining 59.2 and 14.7% of the variance for dominant genera, respectively (Figure 7B). The envfit function showed that environmental variables explained 64.6 and 71.5% of the differences in dominant genera at different growth stages, respectively (Figures 7C,D). SOC was the primary environmental variable affecting the dominant genera at elongation, whereas TN was the primary variable affecting the dominant genera at grain filling (Figures 7C,D).

## 4 Discussion

### 4.1 Crop stage affects the AMF composition

The change in the dominant genera during crop growth affected the community composition of AMF. In this study, the change of dominant genera, including *Claroideoglomus*, *Funneliformis*, *Rhizophagus*, *Entrophospora*, and *Glomus*, altered the community composition of AMF. In addition, our co-occurrence network demonstrated that changes in network complexity could also explain seasonal changes in AMF community structure. The change in keystone OTUs (belongs to the dominant genera *Glomus*, *Claroideoglomus*, and *Rhizophagus*) related to winter wheat growth stage further influenced AMF community composition. In addition, we also found that the relationships between dominant genera (negative relationship: *Claroideoglomus*, *Funneliformis*; positive relationship: *Rhizophagus*, *Entrophospora*, *Glomus*, and *Septoglomus*) and plant biomass altered AMF community composition. This means that growth dynamics could have mediated the relationship between AMF community structure and tillage practices in our study.

Soil nutrients alter AMF communities at different crop stages (Ma et al., 2019). In our study, the mycorrhizal colonization at grain filling was significantly more advanced than elongation (Supplementary Figure S1). A higher mycorrhizal infection could help plants increase their uptake of nutrients (e.g., N and P), thereby promoting plant growth (Gu et al., 2020; Liu et al., 2022). As a result, the change in wheat yield during different crop stages resulted in alterations in AMF communities.

Our results were consistent with previous studies (Abu-Elsaoud et al., 2017), in which the AMF was able to deliver more nutrients during grain filling due to the high nutrient requirement. In addition, MRT results also indicated that soil nitrogen utilization efficiency (TN and NO_3_^−^) played an important role in determining AMF community composition. Moreover, carbon requirements and photosynthetic capacity also affect the community composition of AMF (Louise et al., 2007). Previous studies have shown that the photosynthetic carbon allocation to soil decreased from approximately 10% at elongation to 5% at grain filling in wheat (Sun et al., 2018). In our study, the change of SOC from the elongation stage to the grain filling stage indicated that soil organic carbon affected the AMF community composition. Therefore, the higher SOC at elongation than at grain filling led to the change in the community composition of AMF in our study.

### 4.2 Long-term tillage shifts AMF composition

Long-term conservation tillage (e.g., NT or SS) increased root mycorrhizal colonization. Our results support that the formation of a high colonization rate is linked to plants with rich roots and rapid growth (Ma et al., 2019). Increased contact between roots and AMF propagules under NT or SS can enhance the ability of plants to provide photosynthetic products to fungi, such as grass root systems. Moreover, the NT or SS did not disrupt or weakly interfere with the hyphal networks and dilution of AMF propagules (Kabir, 2005), which is more beneficial to the symbiosis between plants and AMF. We found that the AMF diversity was significantly increased under NT compared to conventional tillage. The results are in agreement with those of previous studies, in which NT versus conventional tillage markedly changed Chao 1, observed species, and PD_whole of AMF at elongation in a greenhouse experiment in Chile (Aguilera et al., 2021), a field experiment in north China (Hu et al., 2015), and a field experiment in Spain (Gu et al., 2020).

In the present study, significant differences in AMF community structure between conservation tillage (NT and SS) and conventional tillage practices can be attributed to changes in the relative abundance of *Claroideoglomus*, *Glomus*, and *Septoglomus*. The genus *Claroideoglomus* can be found in dry or extreme habitats (Al-Yahyáei et al., 2022; Ng et al., 2023). *Claroideoglomus* produces low-radical propagules, which are internal root structures consistent with stress tolerance strategies (Chagnon et al., 2013). As the most abundant genus of AMF across all four treatments, this functional trait may be useful in reducing the impact of abiotic stress factors on the growth of symbiotic plants in conventional tillage practices. In addition, the change in *Glomus* abundance also affects the community composition of AMF through the differences in network complexity. The higher abundance of *Glomus* in NT than in CT is attributed to *Glomus* and may be a legacy of land use due to the tillage history of all experimental plots (Liu et al., 2022). Once conventional tillage practices are suspended, the dominance of highly competitive *Glomus* species may further increase (Sangabriel-Conde et al., 2015). Our results are also in line with a previous study in Mediterranean agroecosystems (Avio et al., 2013), in which *Glomus* species were strong indicators of no-till management and were prevalent in no-till soils.

We observed that wheat biomass and soil properties were negatively correlated with the abundance of *Claroideoglomus*. We attribute the high abundance of *Claroideoglomus* under CT and RT mainly to the adaptation to scarce resources with stress-tolerant life history traits (Silvani et al., 2017). The positive correlation between wheat biomass and *Glomus* abundance indicates that NT and SS can promote soil AMF communities by increasing soil fertility, thereby establishing a mutually beneficial symbiotic relationship with wheat plants and gradually increasing wheat yield (Bunn et al., 2015). In addition, higher TP in the NT and SS treatment also agreed with a previous study where higher soil P content was positively correlated with the relative abundance of *Glomus* and *Septoglomus* (Song et al., 2019; Liu et al., 2022).

## 5 Conclusion

Our results indicate that AMF communities change through the increase or decrease of dominant genera effectively buffering the impact of the ecosystem on environmental changes, thereby improving the overall resilience of the dry environment. No significant difference in AMF community was detected between RT and CT, whereas NT and SS significantly changed the AMF community composition at both growth stages. The CT treatment had higher proportions of the genus *Claroideoglomus*, whereas a higher proportion of the genera *Glomus* and *Septoglomus* was found under NT. Our further study confirmed that NT and SS affected the composition of key genera by changing plant biomass and soil characteristics, thereby affecting the AMF community composition. Our results highlight the importance of wheat biomass and soil properties in modulating the AMF communities. NT and SS are proper tillage practices, which may contribute to the development of sustainable agricultural production in Loess Plateau environments.

## Data availability statement

The data presented in this study are deposited in the online repositories: https://www.ncbi.nlm.nih.gov/sra, accession number PRJNA1090622.

## Author contributions

JiL: Data curation, Methodology, Software, Writing – original draft, Writing – review & editing. LiJ: Data curation, Software, Writing – review & editing. PS: Writing – review & editing. ZA: Writing – review & editing. ZW: Conceptualization, Data curation, Writing – review & editing. ZX: Writing – review & editing. LeJ: Data curation, Software, Writing – review & editing. YY: Data curation, Writing – review & editing. JuL: Data curation, Software, Writing – review & editing. TZ: Data curation, Writing – review & editing. KJ: Project administration, Resources, Writing – review & editing.

## Footnotes

^1^ https://github.com/torognes/vsearch/wiki/VSEARCH-pipeline

^2^ https://gephi.org

## References

[ref1] Abu-Elsaoud A. M. Nafady N. A. Abdel-Azeem A. M. (2017). Arbuscular mycorrhizal strategy for zinc mycoremediation and diminished translocation to shoots and grains in wheat. PLoS One 12:e0188220. doi: 10.1371/journal.pone.0188220, PMID: 29145471 PMC5690681

[ref2] Aguilera P. Romero J. K. Becerra N. Martínez O. Vilela R. Borie F.. (2021). Phenological stages and Aluminum presence influences arbuscular mycorrhizal fungi communities in roots of plant cereals. J. Soil Sci. Plant Nutr. 21, 1467–1473. doi: 10.1007/s42729-021-00453-9

[ref4] Al-Yahyáei M. N. Błaszkowski J. Al-Hashmi H. Al-Farsi K. Al-Rashdi I. Patzelt A.. (2022). From isolation to application: a case study of arbuscular mycorrhizal fungi of the Arabian peninsula. Symbiosis 86, 123–132. doi: 10.1007/s13199-021-00824-x, PMID: 35368327 PMC8933382

[ref5] Augé R. M. Toler H. D. Saxton A. M. (2015). Arbuscular mycorrhizal symbiosis alters stomatal conductance of host plants more under drought than under amply watered conditions: a meta-analysis. Mycorrhiza 25, 13–24. doi: 10.1007/s00572-014-0585-4, PMID: 24831020

[ref6] Avio L. Castaldini M. Fabiani A. Bedini S. Sbrana C. Turrini A.. (2013). Impact of nitrogen fertilization and soil tillage on arbuscular mycorrhizal fungal communities in a Mediterranean agroecosystem. Soil Biol. Biochem. 67, 285–294. doi: 10.1016/j.soilbio.2013.09.005

[ref7] Balota E. L. Machineski O. Honda C. Yada I. F. U. Barbosa G. M. C. Nakatani A. S.. (2016). Response of arbuscular mycorrhizal fungi in different soil tillage systems to long-term swine slurry application. L. Degrad. Dev. 27, 1141–1150. doi: 10.1002/ldr.2304

[ref9] Blois J. L. Williams J. W. Fitzpatrick M. C. Jackson S. T. Ferrier S. (2013). Space can substitute for time in predicting climate change effects on biodiversity. Proc. Natl. Acad. Sci. USA 110, 9374–9379. doi: 10.1073/pnas.1220228110, PMID: 23690569 PMC3677423

[ref10] Bowles T. M. Jackson L. E. Loeher M. Cavagnaro T. R. (2017). Ecological intensification and arbuscular mycorrhizas: a meta-analysis of tillage and cover crop effects. J. Appl. Ecol. 54, 1785–1793. doi: 10.1111/1365-2664.12815

[ref9001] Bunn R. A. Ramsey P. W. Lekberg Y. van der Heijden M. (2015). Do native and invasive plants differ in their interactions with arbuscular mycorrhizal fungi? A meta‐analysis. J. Ecol. 103, 1547–1556. doi: 10.1111/1365-2745.12456

[ref11] Carballar-Hernández S. Hernández-Cuevas L. V. Montaño N. M. Larsen J. FerreraCerrato R. Taboada-Gaytán O. R.. (2017). Native communities of arbuscular mycorrhizal fungi associated with *Capsicum annuum L.* respond to soil properties and agronomic management under field conditions. Agric. Ecosyst. Environ. 245, 43–51. doi: 10.1016/j.agee.2017.05.004

[ref12] Chagnon P. Bradley R. L. Maherali H. Klironomos J. N. (2013). A trait-based framework to understand life history of mycorrhizal fungi. Trends Plant Sci. 18, 484–491. doi: 10.1016/j.tplants.2013.05.001, PMID: 23756036

[ref13] Chen H. Liang Q. Gong Y. Kuzyakov Y. Fan M. Plante A. F. (2019). Reduced tillage and increased residue retention increase enzyme activity and carbon and nitrogen concentrations in soil particle size fractions in a long-term field experiment on loess plateau in China. Soil Tillage Res. 194:104296. doi: 10.1016/j.still.2019.104296

[ref14] Curaqueo G. Barea J. M. Acevedo E. Rubio R. Cornejo P. Borie F. (2011). Effects of different tillage system on arbuscular mycorrhizal fungal propagules and physical properties in a Mediterranean agroecosystem in Central Chile. Soil Tillage Res. 113, 11–18. doi: 10.1016/j.still.2011.02.004

[ref15] Edwards J. Johnson C. Santos-Medellín C. Lurie E. Podishetty N. K. Bhatnagar S.. (2015). Structure, variation, and assembly of the root-associated microbiomes of rice. Proc. Natl. Acad. Sci. USA 112, E911–E920. doi: 10.1073/pnas.1414592112, PMID: 25605935 PMC4345613

[ref16] Gu S. Wu S. Guan Y. Zhai C. Zhang Z. Bello A.. (2020). Arbuscular mycorrhizal fungal community was affected by tillage practices rather than residue management in black soil of Northeast China. Soil Tillage Res. 198:104552. doi: 10.1016/j.still.2019.104552

[ref17] Hamonts K. Trivedi P. Garg A. Janitz C. Grinyer J. Holford P.. (2018). Field study reveals core plant microbiota and relative importance of their drivers. Environ. Microbiol. 20, 124–140. doi: 10.1111/1462-2920.14031, PMID: 29266641

[ref18] Hu J. Yang A. Zhu A. Wang J. Dai J. Wong M. H.. (2015). Arbuscular mycorrhizal fungal diversity, root colonization, and soil alkaline phosphatase activity in response to maize-wheat rotation and no-tillage in North China. J. Microbiol. 53, 454–461. doi: 10.1007/s12275-015-5108-2, PMID: 26115994

[ref19] Jansa J. Mozafar A. Kuhn G. Anken T. Ruh R. Sanders I. R.. (2003). Soil tillage affects the community structures of mycorrhizal fungi in maize roots. Ecol. Appl. 13, 1164–1176. doi: 10.1890/1051-0761(2003)13[1164:statcs]2.0.co;2

[ref20] Jia L. Wang Z. Ji L. De Neve S. Struik P. C. Yao Y.. (2022). Keystone microbiome in the rhizosphere soil reveals the effect of long-term conservation tillage on crop growth in the Chinese loess plateau. Plant Soil 473, 457–472. doi: 10.1007/s11104-022-05297-5

[ref21] Jin K. De Neve S. Moeskops B. Lu J. J. Zhang J. Gabriels D.. (2008). Effects of different soil management practices on winter wheat yield and N losses on a dryland loess soil in China. Soil Res. 46, 455–463. doi: 10.1071/sr07134

[ref22] Jin K. Sleutel S. Buchan D. De Neve S. Cai D. X. Gabriels D.. (2009). Changes of soil enzyme activities under different tillage practices in the Chinese loess plateau. Soil Tillage Res. 104, 115–120. doi: 10.1016/j.still.2009.02.004

[ref24] Kabiri V. Raiesi F. Ghazavi M. A. (2016). Tillage effects on soil microbial biomass, SOM mineralization and enzyme activity in a semi-arid Calcixerepts. Agric. Ecosyst. Environ. 232, 73–84. doi: 10.1016/j.agee.2016.07.022

[ref23] Kabir Z. (2005). Tillage or no-tillage: impact on mycorrhizae. Can. J. Plant Sci. 85, 23–29. doi: 10.4141/p03-160

[ref25] Koljalg U. Nilsson R. H. Abarenkov K. Tedersoo L. Taylor A. F. S. Bahram M.. (2013). Towards a unified paradigm for sequence-based identification of fungi. Mol. Ecol. 22, 5271–5277. doi: 10.1111/mec.12481, PMID: 24112409

[ref26] Lavergne C. Bovio-Winkler P. Etchebehere C. Garcia-Gen S. (2020). Towards centralized biogas plants: co-digestion of sewage sludge and pig manure maintains process performance and active microbiome diversity. Bioresour. Technol. 297:122442. doi: 10.1016/j.biortech.2019.122442, PMID: 31780241

[ref27] Liu C. Cui Y. M. Li X. A. Yao M. J. (2021). Microeco: an R package for data mining in microbial community ecology. FEMS Microbiol. Ecol. 97:fiaa255. doi: 10.1093/femsec/fiaa255 33332530

[ref28] Liu W. Ma K. Wang X. Wang Z. Negrete-Yankelevich S. (2022). Effects of no-tillage and biologically-based organic fertilizer on soil arbuscular mycorrhizal fungal communities in winter wheat field. Appli. Soil Ecol. 178:104564. doi: 10.1016/j.apsoil.2022.104564

[ref29] Louise M. Egerton-Warburto N. C. J. Allen E. B. (2007). Mycorrhizal community dynamics following nitrogen fertilization: a cross-site test in five grasslands. Ecol. Monogr. 77, 527–544. doi: 10.1890/06-1772.1

[ref9002] Lupatini M. Suleiman A. K. A. Jacques R. J. S. Antoniolli Z. I. de Siqueira F. A. Kuramae E. E.. (2014). Network topology reveals high connectance levels and few key microbial genera within soils. Front. Environ. Sci. 2:10. doi: 10.3389/fenvs.2014.00010

[ref30] Ma X. Luo W. Li J. Wu F. (2019). Arbuscular mycorrhizal fungi increase both concentrations and bioavailability of Zn in wheat (*Triticum aestivum* L) grain on Zn-spiked soils. Appl. Soil Ecol. 135, 91–97. doi: 10.1016/j.apsoil.2018.11.007

[ref9003] McGonigle T. P. Miller M. H. Evans D. G. Fairchild G. L. Swan J. A. (1990). A new method which gives an objective measure of colonization of roots by vesicular-arbuscular mycorrhizal fungi. New Phytol. 115, 495–501. doi: 10.1111/j.1469-8137.1990.tb00476.x 33874272

[ref31] Merryweather J. Fitter A. (1998). The arbuscular mycorrhizal fungi of *Hyacinthoides nonscripta* II. Seasonal and spatial patterns of fungal populations. New Phytol. 138, 131–142. doi: 10.1046/j.1469-8137.1998.00889.x

[ref9004] Nelson D. W. Sommers L. E. (1983). Total carbon, organic carbon, and organic matter. Methods of soil analysis: Part 2 chemical and microbiological properties, 9, 539–579. doi: 10.2136/sssabookser5.3.c34

[ref32] Ng A. Wilson B. A. L. Frew A. (2023). Belowground crop responses to root herbivory are associated with the community structure of native arbuscular mycorrhizal fungi. Appl. Soil Ecol. 185:104797. doi: 10.1016/j.apsoil.2022.104797

[ref33] Oehl F. Koch B. (2018). Diversity of arbuscular mycorrhizal fungi in no-till and conventionally tilled vineyards. J. Appl. Bot. Food Qual. 91, 56–60. doi: 10.5073/JABFQ.2018.091.008

[ref34] Panneerselvam P. Kumar U. Senapati A. Parameswaran C. Anandan A. Kumar A.. (2020). Influence of elevated CO_2_ on arbuscular mycorrhizal fungal community elucidated using illumina MiSeq platform in subhumid tropical paddy soil. Appl. Soil Ecol. 145:103344. doi: 10.1016/j.apsoil.2019.08.006

[ref35] Säle V. Aguilera P. Laczko E. Mäder P. Berner A. Zihlmann U.. (2015). Impact of conservation tillage and organic farming on the diversity of arbuscular mycorrhizal fungi. Soil Biol. Biochem. 84, 38–52. doi: 10.1016/j.soilbio.2015.02.005

[ref36] Sangabriel-Conde W. Maldonado-Mendoza I. E. Mancera-Lopez M. E. Cordero-Ramírez J. D. Trejo-Aguilar D. Negrete-Yankelevich S. (2015). Glomeromycota associated with Mexican native maize landraces in Los Tuxtlas, Mexico. Appl. Soil Ecol. 87, 63–71. doi: 10.1016/j.apsoil.2014.10.017

[ref37] Schalamuk S. Velásquez H. Chidichimo H. Cabello M. (2004). Effect of no-till and conventional tillage on mycorrhizal colonization in spring wheat. Bol. Soc. Argent. Bot. 18, 13–20. doi: 10.22370/bolmicol.2003.18.0.375

[ref38] Schnoor T. K. Lekberg Y. Rosendahl S. Olsson P. A. (2011). Mechanical soil disturbance as a determinant of arbuscular mycorrhizal fungal communities in semi-natural grassland. Mycorrhiza 21, 211–220. doi: 10.1007/s00572-010-0325-3, PMID: 20593293

[ref39] Silvani V. A. Colombo R. P. Scorza M. V. Bidondo L. F. Rothen C. P. Scotti A.. (2017). Arbuscular mycorrhizal fungal diversity in high-altitude hypersaline Andean wetlands studied by 454-sequencing and morphological approaches. Symbiosis 72, 143–152. doi: 10.1007/s13199-016-0454-3

[ref40] Singh K. Mishra A. K. Singh B. Singh R. P. Patra D. D. (2016). Tillage effects on crop yield and physicochemical properties of sodic soils. L. Degrad. Dev. 27, 223–230. doi: 10.1002/ldr.2266

[ref41] Soil Survey Staff, (2003). Keys to soil taxonomy, 9th USDA Handbook, NRCS, Washington, DC

[ref42] Song J. Han Y. Bai B. Jin S. He Q. Ren J. (2019). Diversity of arbuscular mycorrhizal fungi in rhizosphere soils of the Chinese medicinal herb *Sophora flavescens* ait. Soil Tillage Res. 195:104423. doi: 10.1016/j.still.2019.104423

[ref43] Song Y. N. Zhang F. S. Marschner P. Fan F. L. Gao H. M. Bao X. G.. (2007). Effect of intercropping on crop yield and chemical and microbiological properties in rhizosphere of wheat (*Triticum aestivum L.*), maize (*Zea mays L.*), and faba bean (*Vicia faba L.*). Biol. Fertil. Soils 43, 565–574. doi: 10.1007/s00374-006-0139-9

[ref45] Sunagawa S. Coelho L. P. Chaffron S. Kultima J. R. Labadie K. Salazar G.. (2015). Structure and function of the global ocean microbiome. Science 348:1261359. doi: 10.1126/science.1261359 25999513

[ref44] Sun Z. Chen Q. Han X. Bol R. Qu B. Meng F. (2018). Allocation of photosynthesized carbon in an intensively farmed winter wheat– soil system as revealed by ^14^CO_2_ pulse labelling. Sci. Rep. 8, 1–10. doi: 10.1038/s41598-018-21547-y, PMID: 29453440 PMC5816614

[ref46] Suzuki K. Takahashi K. Harada N. (2020). Evaluation of primer pairs for studying arbuscular mycorrhizal fungal community compositions using a MiSeq platform. Biol. Fertil. Soils 56, 853–858. doi: 10.1007/s00374-020-01431-6

[ref48] Vance E. D. Brookes P. C. Jenkinson D. (1987). An extraction method for measuring microbial biomass carbon. Soil Biol. Biochem. 19, 703–707. doi: 10.1016/0038-0717(87)90052-6

[ref47] Van Geel M. Busschaert P. Honnay O. Lievens B. (2014). Evaluation of six primer pairs targeting the nuclear rRNA operon for characterization of arbuscular mycorrhizal fungal (AMF) communities using 454 pyrosequencing. J. Microbiol. Methods 106, 93–100. doi: 10.1016/j.mimet.2014.08.006, PMID: 25173951

[ref49] Walder F. van der Heijden M. (2015). Regulation of resource exchange in the arbuscular mycorrhizal symbiosis. Nat. Plants 1:15159. doi: 10.1038/nplants.2015.159 27251530

[ref52] Wang S. L. Wang H. Muhammad B. H. Zhang Q. Yu Q. Wang R.. (2020). No-tillage and subsoiling increased maize yields and soil water storage under varied rainfall distribution: a 9-year site-specific study in a semiarid environment. Field Crop Res. 255, 107867–107869. doi: 10.1016/j.fcr.2020.107867

[ref51] Wang W. X. Shi J. C. Xie Q. J. Jiang Y. N. Yu N. Wang E. T. (2017). Nutrient exchange and regulation in arbuscular mycorrhizal symbiosis. Mol. Plant 10, 1147–1158. doi: 10.1016/j.molp.2017.07.012 28782719

[ref50] Wang Z. Li Y. Li T. Zhao D. Liao Y. (2020). Tillage practices with different soil disturbance shape the rhizosphere bacterial community throughout crop growth. Soil Tillage Res. 197:104501. doi: 10.1016/j.still.2019.104501

[ref9005] Xia Q. Liu X. Gao Z. Wang J. Yang Z. (2020). Responses of rhizosphere soil bacteria to 2-year tillage rotation treatments during fallow period in semiarid southeastern Loess Plateau. Peerj 8:e8853. doi: 10.7717/peerj.8853 32411509 PMC7207221

[ref53] Xiao D. Xiao S. Ye Y. Zhang W. He X. Wang K. (2019). Microbial biomass, metabolic functional diversity, and activity are affected differently by tillage disturbance and maize planting in a typical karst calcareous soil. J. Soils Sediments 19, 809–821. doi: 10.1007/s11368-018-2101-5

[ref9006] Xue L. Z. Khan S. Sun M. Anwar S. Ren A. X. Gao Z. Q.. (2019). Effects of tillage practices on water consumption and grain yield of dryland winter wheat under different precipitation distribution in the loess plateau of China. Soil Tillage Res. 191, 66–74. doi: 10.1016/j.still.2019.03.014

[ref55] Yang Y. Song Y. Scheller H. V. Ghosh A. Bai Y. Chen H.. (2015). Community structure of arbuscular mycorrhizal fungi associated with *Robinia pseudoacacia* in uncontaminated and heavy metal contaminated soils. Soil Biol. Biochem. 86, 146–158. doi: 10.1016/j.soilbio.2015.03.018

[ref54] Yan S. S. Song J. M. Fan J. S. Yan C. Dong S. K. Ma C. M.. (2020). Changes in soil organic carbon fractions and microbial community under rice straw return in Northeast China. Glob. Ecol. Conserv. 22, e00962–e00912. doi: 10.1016/j.gecco.2020.e00962

[ref56] Zhang S. Li Q. Lü Y. Sun X. Jia S. Zhang X.. (2015). Conservation tillage positively influences the microflora and microfauna in the black soil of Northeast China. Soil Tillage Res. 149, 46–52. doi: 10.1016/j.still.2015.01.001

[ref57] Zuber S. M. Behnke G. D. Nafziger E. D. Villamil M. B. (2015). Crop rotation and tillage effects on soil physical and chemical properties in Illinois. Agron. J. 107, 971–978. doi: 10.2134/agronj14.0465

