# Supplementary material for: Plant and soil responses to tillage practices change arbuscular mycorrhizal fungi populations during crop growth
Source: Front Microbiol. 2024 Apr 8;15:1394104. doi: 10.3389/fmicb.2024.1394104 (PMC11034428; doi:10.3389/fmicb.2024.1394104)
Supplement: Supplementary file 1 [file Data_Sheet_1.docx]

Supplementary Material

- 1. **Supplementary Figures**

**
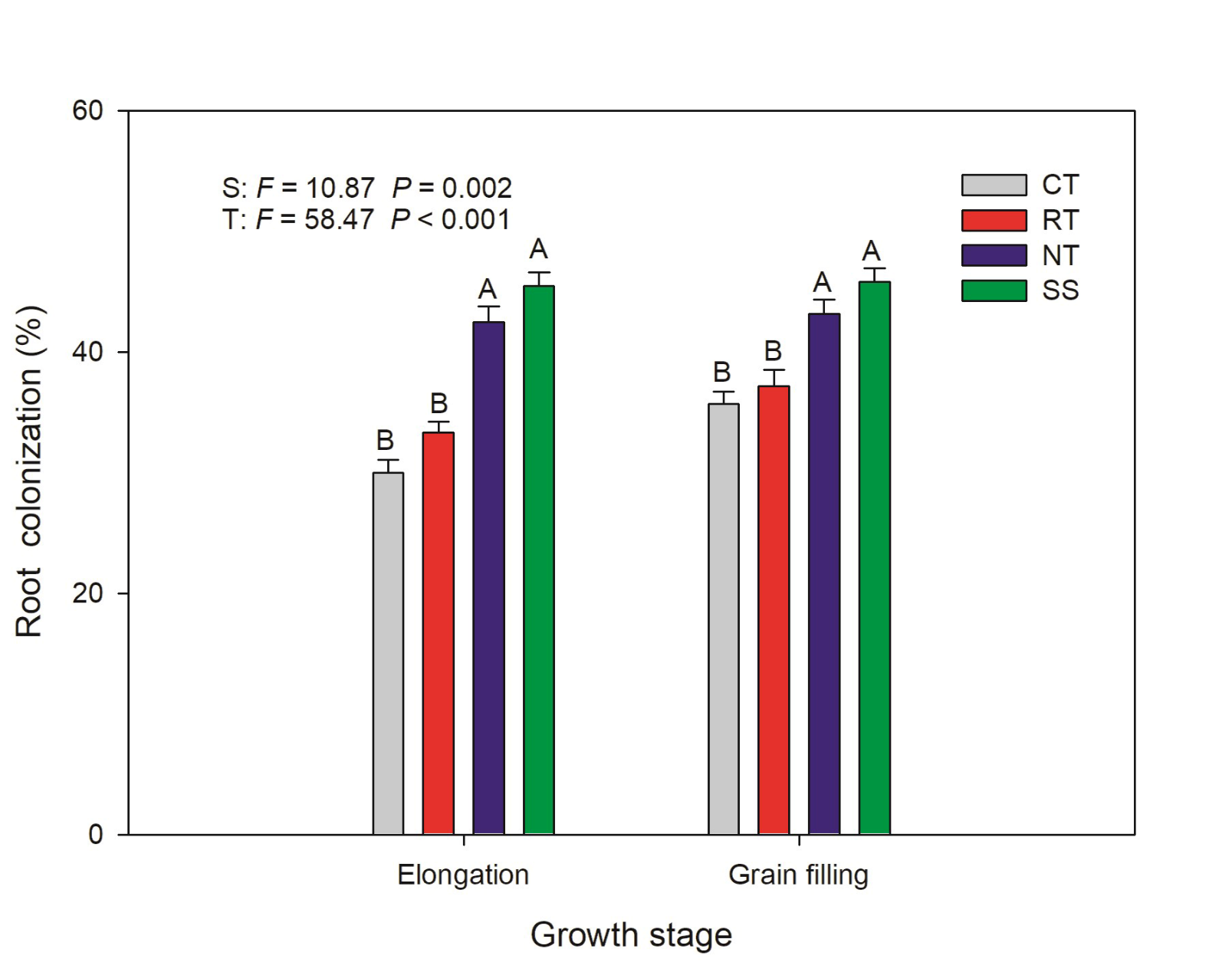
**

**Supplementary Figure 1.** Arbuscular mycorrhizal fungal root colonization among different tillage practices under two growth stages. Shared letters denote no significant difference among different tillage practices according to Tukey HSD test at *P* < 0.05. CT: conventional tillage (n = 6), NT: no tillage with mulch (n = 6), RT: reduced tillage (n = 6), SS subsoiling with mulch (n = 6).

- 1. **Supplementary Tables**

**Supplementary Table 1.** The key OTU selected by the greatest values of Betweenness Centrality (BC) found in each of the sampling sites.

| **Id** | ***BC*** | ***CC*** | **Abundance**  **(%)** | **Taxonomy** |
| --- | --- | --- | --- | --- |
| **Elongation stage** | | | | |
| OTU_120 | 95.366667 | 0.511111 | 0.05 | *Mucoromycota*; *Glomeromycetes*; *Glomerales*; *Glomeraceae*; *Glomus*; *Glomus_aggregatum* |
| OTU_231 | 95.133333 | 0.46 | 0.08 | *Mucoromycota; Glomeromycetes; Glomerales; Claroideoglomeraceae; Claroideoglomus; Claroideoglomus_drummondii* |
| OTU_51 | 83.666667 | 0.45098 | 2.38 | *Mucoromycota; Glomeromycetes; Glomerales; Glomeraceae; Glomus; Glomus_aggregatum* |
| OTU_122 | 63.133333 | 0.5 | 1.29 | *Mucoromycota; Glomeromycetes; Glomerales; Glomeraceae; Glomus; Glomus_aggregatum* |
| OTU_67 | 60.0 | 0.343284 | 0.14 | *Mucoromycota; Glomeromycetes; Glomerales; Glomeraceae; Glomus; Glomus_sp._8_ZHNL-2013o* |
| **Grain filling stage** | | | | |
| OTU_28 | 46.833333 | 0.666667 | 0.56 | *Mucoromycota; Glomeromycetes; Glomerales; Glomeraceae; Rhizophagus; Rhizophagus_intraradices* |
| OTU_181 | 33.0 | 0.56 | 0.02 | *Mucoromycota; Glomeromycetes; Glomerales; Glomeraceae; unidentified; Glomeraceae_sp.* |
| OTU_8 | 33.0 | 0.538462 | 0.99 | *Mucoromycota; Glomeromycetes; Glomerales; Glomeraceae; Glomus; Glomus_sp._7_ZHNL-2013a* |
| OTU_19 | 25.0 | 0.50 | 0.05 | *Mucoromycota; Glomeromycetes; Glomerales; Glomeraceae; Glomus; Glomus_sp._8_SUN-2011* |
| OTU_140 | 24.666667 | 0.56 | 0.05 | *Mucoromycota; Glomeromycetes; Glomerales; Glomeraceae; Glomus; Glomus_aggregatum* |
| OTU_175 | 24.0 | 0.4 | 0.03 | *Mucoromycota; Glomeromycetes; Glomerales; Glomeraceae; Glomus; Glomus_aggregatum* |
